# Mark Hallett (1943–2025): a personal recollection

**DOI:** 10.3389/fneur.2025.1747674

**Published:** 2026-01-08

**Authors:** Alberto Albanese

**Affiliations:** 1 Department of Neuroscience, Catholic University of the Sacred Heart, Milano, Italy; 2 Department of Neurology, Fondazione IRCCS Istituto Neurologico Nazionale Casimiro Mondino, Pavia, Italy

**Keywords:** myoclonus, dystonia, movement disorders, neurophysiology, history, biography

Mark was special to me. I have always appreciated his approach to science and his openness. I have never seen him upset or polemical, always modest in attitude and at the same time brilliantly political and well-balanced. We never had an argument and I always considered his opinions thoughtful and enlightening. While going out to dinner after a day in a conference, he typically wore a light raincoat with his rain hat in a pocket. His gait was a phenomenological certainty to me and we typically discussed the day along our way to the restaurant. We made jokes on how quick he was in replying to emails, usually the first to reply, within seconds, even at improbable times as he was often traveling across different time zones.

Mark had been one of the early visiting researchers with David Marsden at the newly established lab in Windsor Walk. He had been trained in neurophysiology at Harvard and planned to study the physiology of normal rapid movements when he joined Marsden in 1975. He initially participated to the research projects on reflex responses that David had carried out with Pat Merton and H. B. Morton (the “3M” experiments), but soon David proposed him to study myoclonus and assembled a research team with Mark as neurophysiologist, Peter Jenner as pharmacologist and David Chadwick as clinical evaluator. This research led to a bunch of important publications on myoclonus (1–4). Mark maintained his interest in myoclonus throughout his scientific career, until recently he coauthored the new classification of myoclonus that appeared this year (5). Mark was special to all and to David Marsden as well. At age 60, in 1998, David took a sabbatical year at Mark's lab in Bethesda, Maryland, and there he died unexpectedly only 4 weeks into his visit.

Mark was also interested in the pathophysiology of dystonia a theme that brought us close to each other. We started collaborating when I was asked to organize the 2008 Toxin conference in Baveno. The informal toxin group held a meeting every 3 years and the local organizer bore all the responsibility. The scientific committee was composed of basic scientists and clinicians. In that committee, I partnered particularly with Mark and Joe Jankovic. The conference was a great success with a positive economic balance and I proposed using the proceeds to establish a new scientific society. Mark introduced me to an American lawyer and the three of us met regularly for about a year to finalize the new bylaws. As congress chair, I was paying with the conference account and the lawyer sent his bills to my department fax. The secretary became suspicious of seeing so many bills from a law firm in the United States and started asking me if I had caused any trouble there. These bills drained the conference account just after the new association, named International Neurotoxin Association (INA), was established in 2010. We had made it!

I like to remember Mark sitting relaxed under a large pop art picture. We were in Miami in January 2011 and the INA bylaws had just been finalized (Figure 1). Later Mark asked the same lawyer to prepare the bylaws for the Functional Movement Disorders Society he founded in 2020. He told me that the INA bylaws served as a useful draft to save time and money. Our mutual confidence grew over time and all the Toxins conferences marked positive moments of scientific and personal interactions (Figure 2).

**Figure 1:**
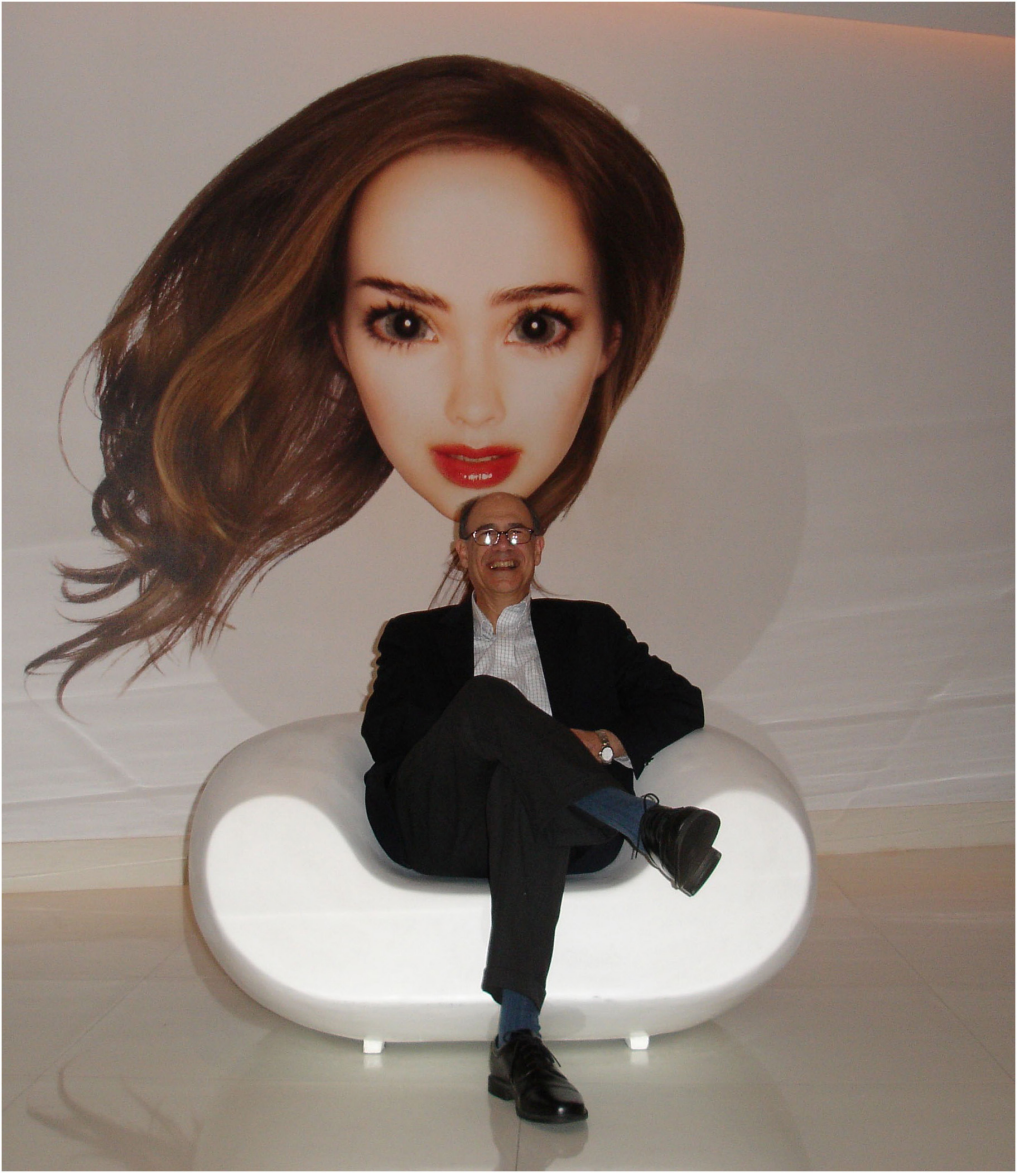
Mark Hallett pictured by Alberto Albanese in January 2011 in Miami.

**Figure 2:**
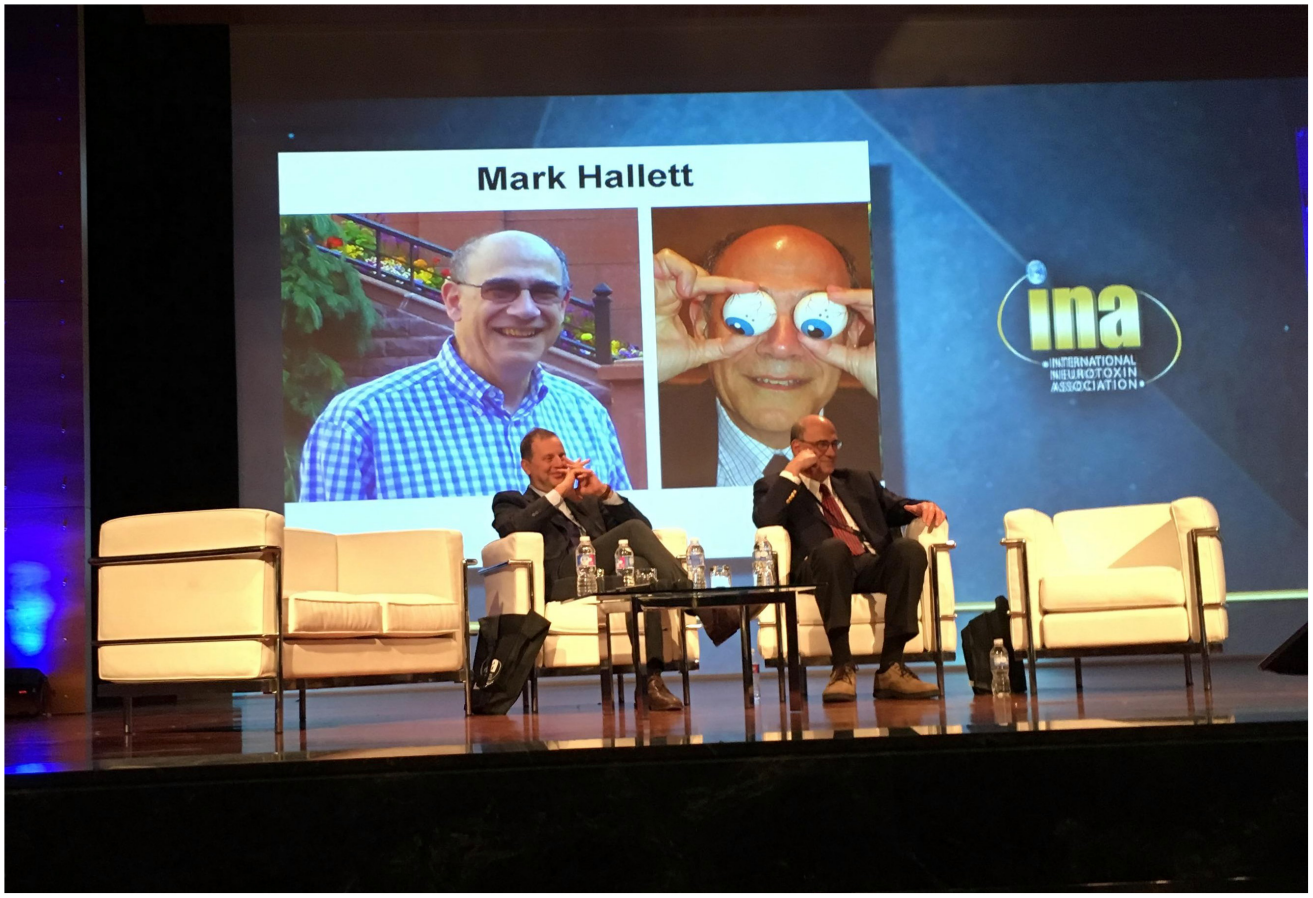
Alberto Albanese and Mark Hallett in January 2017 at the International Neurotoxin Association congress in Madrid.

In 2011 we collaborated to the first consensus on dystonia. This was a challenging and very innovative experience that started as a classical consensus meeting and unpredictably evolved into an innovative double-axis classification, which was published in 2013 (6). This effort was intellectually challenging and Mark liked it so much that he also participated on two other consensus committees adopting similar classification schemes, one on tremor published in 2018 (7) and another on myoclonus published in 2025 (5). He poured his clinical and neurophysiological skills into these efforts. Indeed, Mark had two main scientific sides: he loved neurophysiology, but also loved and practiced clinical neurology. This combination was his intellectual fuel, and his interventions typically combined phenomenology and pathophysiology. He was particularly interested in all types of hyperkinetic movements and also in the pathophysiology of hypokinetic states. Mark was also part of the dystonia revision consensus published in 2025 (8). There, he proposed to include a third axis on pathophysiology. He was puzzled by the idea of a third axis and we had several discussions on this possible option. Could we include pathophysiology in the revision of the dystonia classification? We performed several simulations until we eventually decided that the times were not ready for this addition. We knew that Mark was considering a possible inclusion in years to come, as he wanted to accommodate pathophysiology into a comprehensive classification system.

When his daughter came to Florence, where she stayed for about a year, Mark increased his visits to Italy and his wife Judith came along more often. I had a sister living in Florence, who provided some local support, but I had few occasions to go there. Mark had hosted so many international researchers and was so internationally renowned to receive continuous invitations. He was happy to travel and knew virtually anyone. It was easier to meet him internationally, as he was a true globetrotter. Almost anywhere he had fellows who regularly invited him. Mark not only knew many places, but also mastered the charming and attractive details of whereabouts across the continents. Traveling with him was an enjoyable experience.

Our last e-mail exchanges were in August. His messages had become more essential, with shorter answers. I was so happy he was active, although I knew his tumor was unappealable. I will miss Mark a lot. I remember sitting with him in his studio in the lower floor of his home, where his typical gentle laugh resounded: it allowed him to take few instants of reflection before replying. With his passing away I feel that some of my strength and certainty have gone. I will keep looking for his sincere and empathic smile at next conference, for his raincoat and unmistakable proximity.

I wish him a safe journey up there.

## In memoriam

This paper is dedicated to the memory of Mark Hallett (1943–2025).
